# A Systematic Review of Predictor Composition, Outcomes, Risk of Bias, and Validation of COVID-19 Prognostic Scores

**DOI:** 10.1093/cid/ciad618

**Published:** 2023-10-25

**Authors:** Katharina S Appel, Ramsia Geisler, Daniel Maier, Olga Miljukov, Sina M Pütz, J Janne Vehreschild

**Affiliations:** Department II of Internal Medicine, Hematology/Oncology, Goethe University Frankfurt, Frankfurt am Main, Germany; Department II of Internal Medicine, Hematology/Oncology, Goethe University Frankfurt, Frankfurt am Main, Germany; Department II of Internal Medicine, Hematology/Oncology, Goethe University Frankfurt, Frankfurt am Main, Germany; German Cancer Consortium (DKTK), Partner Site Frankfurt/Mainz and German Cancer Research Center (DKFZ), Heidelberg, Germany; Institute of Clinical Epidemiology and Biometry, University of Würzburg, Würzburg, Germany; University of Cologne, Faculty of Medicine and University Hospital Cologne, Department I of Internal Medicine, Center for Integrated Oncology Aachen Bonn Cologne Duesseldorf, Cologne, Germany, University of Cologne; Department II of Internal Medicine, Hematology/Oncology, Goethe University Frankfurt, Frankfurt am Main, Germany; University of Cologne, Faculty of Medicine and University Hospital Cologne, Department I of Internal Medicine, Cologne, Germany; German Centre for Infection Research (DZIF), partner site Bonn-Cologne, Cologne, Germany

**Keywords:** COVID-19, scores, prediction models, predictors, pandemic preparedness

## Abstract

**Background:**

Numerous prognostic scores have been published to support risk stratification for patients with coronavirus disease 2019 (COVID-19).

**Methods:**

We performed a systematic review to identify the scores for confirmed or clinically assumed COVID-19 cases. An in-depth assessment and risk of bias (ROB) analysis (*Prediction model Risk Of Bias ASsessment Tool* [PROBAST]) was conducted for scores fulfilling predefined criteria ([I] area under the curve [AUC)] ≥ 0.75; [II] a separate validation cohort present; [III] training data from a multicenter setting [≥2 centers]; [IV] point-scale scoring system).

**Results:**

Out of 1522 studies extracted from MEDLINE/Web of Science (20/02/2023), we identified 242 scores for COVID-19 outcome prognosis (mortality 109, severity 116, hospitalization 14, long-term sequelae 3). Most scores were developed using retrospective (75.2%) or single-center (57.1%) cohorts. Predictor analysis revealed the primary use of laboratory data and sociodemographic information in mortality and severity scores. Forty-nine scores were included in the in-depth analysis. The results indicated heterogeneous quality and predictor selection, with only five scores featuring low ROB. Among those, based on the number and heterogeneity of validation studies, only the 4C Mortality Score can be recommended for clinical application so far.

**Conclusions:**

The application and translation of most existing COVID scores appear unreliable. Guided development and predictor selection would have improved the generalizability of the scores and may enhance pandemic preparedness in the future.

## Teaser

This systematic review of clinical coronavirus disease 2019 (COVID-19) scores revealed a large quantity and heterogeneity of their quality and predictor composition. We exhibit challenges for transferability across care settings. The results may support score implementation and future pandemic preparedness.

The coronavirus disease 2019 (COVID-19) pandemic has created a state of emergency in health systems across the globe [1]. Hospitals were overcrowded with patients and decisions for their management had to be made quickly. At the same time resource constraints limit the treatment of all patients with adequate therapies. Even in 2023, when the pandemic transitioned into an endemic state [2], the dynamically evolving variants of the severe acute respiratory syndrome coronavirus 2 (SARS-CoV-2) still cause severe disease in individuals, regardless of immunity, vaccines, and therapeutic interventions [3, 4], especially when they are of elevated age or have comorbidities [5].

Especially during the first wave of the pandemic, scientists and clinicians rushed their efforts to support decision making, often trying to define thresholds of defined symptoms or scores. Such clinical prognostic scores are derived from models that estimate an individual's probability for a particular condition by combining and weighting predictive factors, mainly in an easy-to-apply manner (eg, additive point systems). Compared to a more complex, information-intense, and accurate (statistical) outcome prediction model, a score is a clinical decision support tool that facilitates fast applicability and unambiguous communication. Clinicians use such scores as “prediction rules” daily to reduce severe outcomes by modifying therapeutic considerations according to given risks [6, 7]. Although clinical judgments remain irreplaceable [5], a score's validity, reliability, and trustworthiness depend on the quality criteria applied during development and adequate validation. Scores can be developed for different scenarios (eg, predicting in-hospital mortality after admission or hospitalization at diagnosis) [7], making their application relevant in different settings.

Although numerous predictive models for COVID-19 were published [8–10], most are of heterogeneous methodological quality or remain unvalidated. The scores were not universally implemented in everyday clinical care and treatment instructions. The current Infectious Diseases Society of America (IDSA) guideline (05/2023) [11] does not recommend a specific tool for outcome prognosis. The World Health Organization's (WHO) guideline on *Therapeutics and COVID-19* (01/2023) [4] reported that reliable tools are needed, especially for using available medication. Although it mentioned the ISARIC's (*International Severe Acute Respiratory and emerging Infection Consortium*) 4C Mortality Score (4C) [12], the “need for better evidence on prognosis” is emphasized [4]. The WHO's *Living Guidance for clinical management of COVID-19* (01/2023) also suggests “clinical judgment […] rather than currently available prediction models” [13]. In summary, evidence for prognostic scores is poor [8–10], and the translation into clinical practice remains elusive. At the same time, the need for reliable stratification tools is emphasized in COVID-19 guidelines [4, 13].

In our systematic review, we focus on the critical appraisal of predictors and the transferability of clinical scores to support implementation in routine care. We aim to identify scores for daily clinical care, provide an effective overview for decision-makers, and pave the way for future pandemic preparedness.

## METHODS

### Systematic Review Question, Inclusion, and Exclusion Criteria

For this systematic review, we identified the COVID-19 prognostic clinical scores developed from the onset of the pandemic. We included original scores designed or modified for the management of COVID-19 based on individual patient data from clinically assumed or confirmed COVID-19 cases. We did not preselect publications on specific patient care levels, timings of predictor measurement, predictor types, or targeted specific outcomes. We excluded regression or other prediction models unsuitable for scoring, predictors based on single observations, scores focusing on specific subpopulations (eg, comorbidities, pharmaceutical trials), and mathematical virus transmission simulations. In the first step, we extracted information from all identified studies (termed “all scores”) that fulfilled the primary inclusion criteria (see Table 1). Second, we selected scores for an in-depth analysis (Level 2 [L2]) based on predefined criteria: (I) area under the curve (AUC) ≥ 0.75, (II) a separate validation cohort, (III) training data from a multicenter setting (≥ 2 centers), and (IV) the result of the score mapped on a point scale (for details see Supplementary Text 1). Only scores fulfilling the L2 criteria were further evaluated for risk of bias (ROB). The other scores were assigned to Level 1 (L1).

**Table 1.** Inclusion and Exclusion Criteria for the Selection of Literature

Inclusion Criteria	Exclusion Criteria
Original publication of a new or modified score developed in the context of COVID-19 In- and outpatients with clinically assumed or confirmed COVID-19 (laboratory tests (polymerase chain reaction, serological test), or clinical assumption or diagnosis) Transparent and reproducible algorithm presented Combination of at least 2 predictors Article written in English or German language	Models built on specific subpopulations (eg, comorbidities, specific pharmaceutical interventions, pregnancy) Models without scoring character or pure presentation as web calculators or nomograms Models of single predictors,^a^ genetic factors, or mental health outcomes (burnout, stress, resilience) (Quantitative) radiologic scores with image processing^a^

Abbreviation: COVID-19, coronavirus disease 2019.

^a^A radiological score without further combination with other clinical predictors was considered a single predictor.

### Data Sources, Search Strategy, and Data Extraction

We searched *MEDLINE* and *Web of Science* on 14 April 2022 and 20 February 2023 using a prespecified search strategy combining domains regarding “COVID-19”, “Prediction”, “Scoring” and “Validation metrics” (Supplementary Text 2). Our processing was based on the *Preferred Reporting Items for Systematic reviews and Meta-Analyses* (PRISMA) guidelines [14]. For the extracted information, see Supplementary Text 3. All literature processing tasks, including screening, data extraction, and ROB assessment were independently performed by two reviewers (K. A., R. G.). In case of disagreement, consensus was reached by discussion.

If not stated otherwise, the unit of analysis was one score per outcome and predictor set. We also provide an overview of external validations identified by an ad hoc search in the same literature retrieval with a reduced set of extraction items.

Extracted AUCs are presented with range or median and interquartile range (IQR); categorical information is reported in absolute numbers and percentages (n (%)). The sample size was evaluated using the (estimated) events per variable (EPV), with low EPVs indicating a higher risk for overfitting (see Supplementary Text 3) [15].

### Outcomes and Categorization of Scores

Based on the identified literature, the following outcomes were present: fatal outcomes (in-hospital mortality, death within specified time intervals), disease severity (classified as composite outcomes, eg, need for mechanical ventilation, intensive care unit [ICU] admission, or death), hospitalization, and the post-COVID condition (PCC). We categorized the scores by the type of outcome and the timing of predictor measurement (Table 2).

**Table 2.** Categories by Timing of Predictor Measurement and Outcome

No.	Category
1	First/early contact with healthcare facility ➝ Fatal outcome
2	First/early contact with healthcare facility ➝ Deterioration (severity, ICU admission, need for mechanical ventilation, respiratory complication, specific organ failures, or fatal outcome as a composite endpoint, etc.)
3	Severe disease or ICU admission ➝ Deterioration or fatal outcome
4	First diagnosis and contact with outpatient healthcare facility ➝ Hospitalization
5	Acute infection ➝ PCC

Contact with healthcare facility also includes hospital or emergency department (ED) admission or the first diagnosis by SARS-CoV-2 testing. Abbreviations: ICU, intensive care unit; PCC, post-COVID-19 condition; SARS-CoV-2, severe acute respiratory syndrome coronavirus 2.

### Risk of Bias Assessment

Flaws in a study's design, conduct, or analysis methods can cause systematic errors (bias) of effect estimates. The *Prediction model Risk Of Bias Assessment Tool* (PROBAST) specifies the adequacy of methods when developing a clinical prediction rule by assessing the ROB within its four subdomains: “participants,” “predictors,” “outcome,” and “analysis.” The ratings “low,” “unclear,” or “high” evaluate the validity of the study and condense to an overall ROB. A “high” rating within at least 1 question or subdomain leads to an overall ROB of “high” [15].

## RESULTS

**Figure 1.**
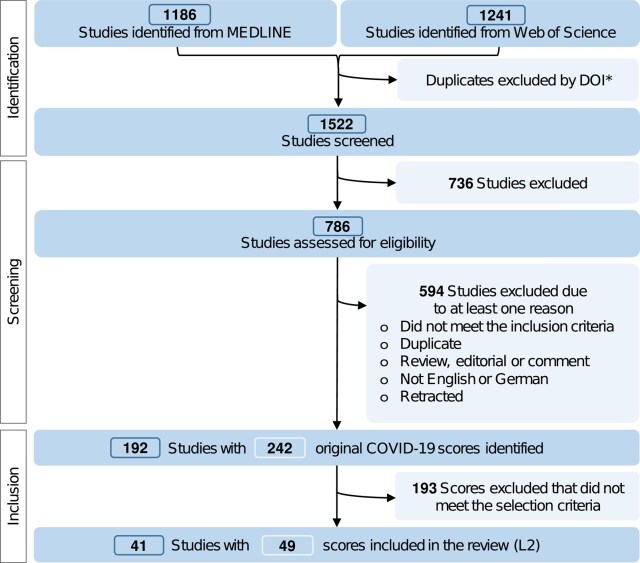
PRISMA flow chart. *Not every study had a DOI or had multiple DOIs. Abbreviations: COVID-19, coronavirus disease 2019; DOI, digital object identifier; PRISMA, Preferred Reporting Items for Systematic reviews and Meta-Analyses.

The PRISMA flow chart (Figure 1) shows the literature evaluation procedure. Of 1522 studies extracted from the database, 242 original COVID-19 scores met the primary inclusion criteria, and 49 met the L2 criteria (details for all scores in Supplementary Table 1 and L2 in Supplementary Table 2). Comparative summary statistics matching this section are presented in Table 3.

**Table 3.** Characteristics of the Included Scores

Characteristics	All	Level	
	N = 242 n (%)^e^	Level 1^d^, N = 193 n (%)^e^	Level 2, N = 49 n (%)^e^
Category			
1 First/early contact with healthcare facility ➝ Fatal outcome	100 (41.3)	79 (40.9)	21 (42.9)
2 First/early contact with healthcare facility ➝ Deterioration	112 (46.3)	94 (48.7)	18 (36.7)
3 Severe disease or ICU admission ➝ Deterioration or fatal outcome	13 (5.4)	12 (6.2)	1 (2.0)
4 First diagnosis and contact with outpatient healthcare facility ➝ Hospitalization	14 (5.8)	5 (2.6)	9 (18.4)
5 Acute infection ➝ PCC	3 (1.2)	3 (1.6)	0 (0.0)
Study design			
Prospective	33 (13.6)	30 (15.5)	3 (6.1)
Retro- and prospective	12 (5.0)	3 (1.6)	9 (18.4)
Retrospective	182 (75.2)	150 (77.7)	32 (65.3)
Unknown	15 (6.2)	10 (5.2)	5 (10.2)
Multicenter design			
≥ 2 centers	103 (42.9)	54 (28.3)	49 (100.0)
Samples size			
Cumulative number of participants ≥1000	87 (36.0)	47 (24.4)	40 (81.6)
Estimated events per variable^a^ (median, IQR)	…	…	15.6 (IQR = [6.6, 267.3])
Health sector			
Hospitals/emergency department	216 (89.6)	182 (94.8)	34 (69.4)
In- or outpatient sites	16 (6.6)	3 (1.6)	13 (26.5)
Outpatient sites	7 (2.9)	5 (2.6)	2 (4.1)
Other	2 (0.8)	2 (1.0)	0 (0.0)
Population			
Patients in the emergency department	25 (10.3)	18 (9.3)	7 (14.3)
Inpatients with severe disease	37 (15.3)	35 (18.1)	2 (4.1)
Inpatients without restriction to specific conditions^b^	158 (65.3)	132 (68.4)	26 (53.1)
Inhabitants of one region	1 (0.4)	1 (0.5)	0 (0.0)
Out- and inpatients	11 (4.5)	2 (1.0)	9 (18.4)
Outpatients	10 (4.1)	5 (2.6)	5 (10.2)
Study/recruitment time			
2020	…	…	38 (77.6)
2020–2021	…	…	4 (8.2)
2020–2022	…	…	7 (14.3)
Country			
China	45 (18.6)	39 (20.2)	6 (12.2)
Italy	25 (10.3)	24 (12.4)	1 (2.0)
United States	33 (13.6)	18 (9.3)	15 (30.6)
Other	139 (57.4)	112 (58.0)	27 (55.1)
Timing of predictor measurement			
Admission to hospital or emergency department	190 (79.8)	159 (84.1)	31 (63.3)
Admission to ICU	7 (2.9)	6 (3.2)	1 (2.0)
SARS-CoV2 testing/diagnosis	13 (5.5)	12 (6.3)	1 (2.0)
Other	28 (11.8)	12 (6.3)	16 (32.7)
Outcomes			
Deterioration (composite, with fatal outcomes)	109 (45.0)	91 (47.2)	18 (36.7)
Fatal outcomes (single endpoint)	116 (47.9)	94 (48.7)	22 (44.9)
Hospitalization	14 (5.8)	5 (2.6)	9 (18.4)
Post-acute COVID syndrome	3 (1.2)	3 (1.6)	0 (0.0)
Handling of missing values			
Any imputation method applied	…	…	19 (38.8)
Multiple imputation	…	…	11 (22.4)
Modeling technique			
(Cox, (Bayesian) Logistic, LASSO) Regression	…	…	41 (83.7)
Machine learning	…	…	2 (4.1)
Mixed methods or other	…	…	6 (12.2)
Validation^c^			
Separate cohort present	138 (57.0)	89 (46.1)	49 (100)
Geographical validation	…	…	10 (20.4)
Temporal validation	…	…	17 (34.7)
Temporal and geographical validation	…	…	7 (14.3)
Random split	…	…	13 (26.5)
Validation with different population characteristics	…	…	1 (2.0)
Independent external validation	…	…	2 (4.1)
Discrimination			
AUC of the strongest validation ≥ 0.75	190 (78.5)	141 (73.1)	49 (100.0)
AUC (median, IQR)	0.83 (IQR = [0.77, 0.90])	0.84 (IQR = [0.77, 0.91])	0.81 (IQR = [0.80, 0.85])
Calibration^c^			
Any method applied	…	…	30 (61.2)
Calibration plot or table	…	…	23 (46.9)
Hosmer-Lemeshow	…	…	12 (24.5)
Application			
Formula	65 (26.9)	65 (33.7)	0 (0.0)
Points-based and formula	172 (71.1)	123 (63.7)	49 (100.0)
Formula	3 (1.2)	3 (1.6)	0 (0.0)
Other	2 (0.8)	2 (1.0)	0 (0.0)

We present n (%) for categorical information and the median (IQR) for continuous information. The column “All” includes all scores fulfilling the a priori inclusion criteria. In contrast, Level 1 merely includes scores that did not fulfill the selection criteria and Level 2 only includes the scores fulfilling the criteria (see Methods section). As a result of two granularity levels of data extraction, some information is only available for Level 2 scores.

Abbreviations: AUC, area under the curve; COVID, coronavirus disease; ICU, intensive care unit; IQR, interquartile range; PCC, post-COVID-19 condition; SARS-CoV-2, severe acute respiratory syndrome coronavirus 2.

^a^Events per variable (EPV) were estimated using the absolute number of candidate predictors. Some studies did not precisely name the number of candidate predictors. To generate assumptions regarding the sample size, we counted predictors indicated as candidates in tables or texts (signed by “∼” in Supplementary Table 2), even though we acknowledge that using the number of regression coefficients instead is more precise [15].

^b^Regarding population characteristics, “severe disease” includes ICU patients and patients with respiratory complications, pneumonia, intubation, or other severe conditions.

^c^Multiple options possible.

^d^Level 1 (L1) includes those scores among “all” scores that did not fulfill the Level 2 selection criteria.

^e^Or median with IQR.

### Data Basis and General Study Characteristics (All Scores)

All studies were published between 2020 and 2023. Most scores were developed based on cohorts with <1000 participants (64.0%) in a retrospective (75.2%) and/or single-center (57.1%) design. Fifty-seven percent of the models were validated in a separate cohort, including random splits, temporal, or geographical (external) validation. The median AUC was 0.83 (IQR = [0.77, 0.90]).

The study populations included hospitalized cases without restriction to specific conditions (65.3%), patients with severe disease (15.3%), or patients admitted to the emergency department (ED) (10.3%). The major timing for prediction was admission to hospital or emergency department (ED) (79.8%). Predicted outcomes (all scores) were mortality (45.0%), severity (as composite endpoints) (47.9%), hospitalization (5.8%), and PCC (1.2%).

**Figure 2.**
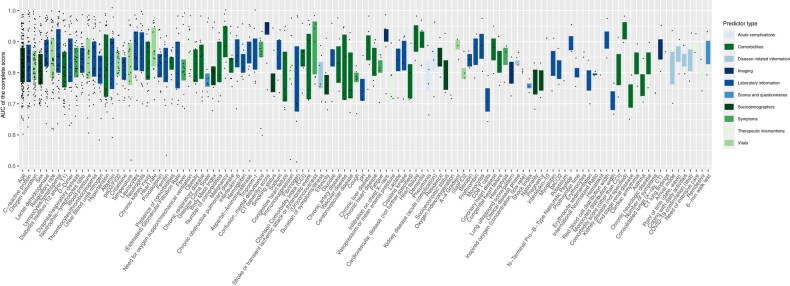
Relationship between predictor frequency within all scores (irrespective of category) and AUC of the overall score. The predictors were grouped by predictor type. Only predictors that were integrated at least twice in a score are presented. Abbreviations: AUC, area under the curve; COVID-19, coronavirus disease 2019.

**Figure 3.**
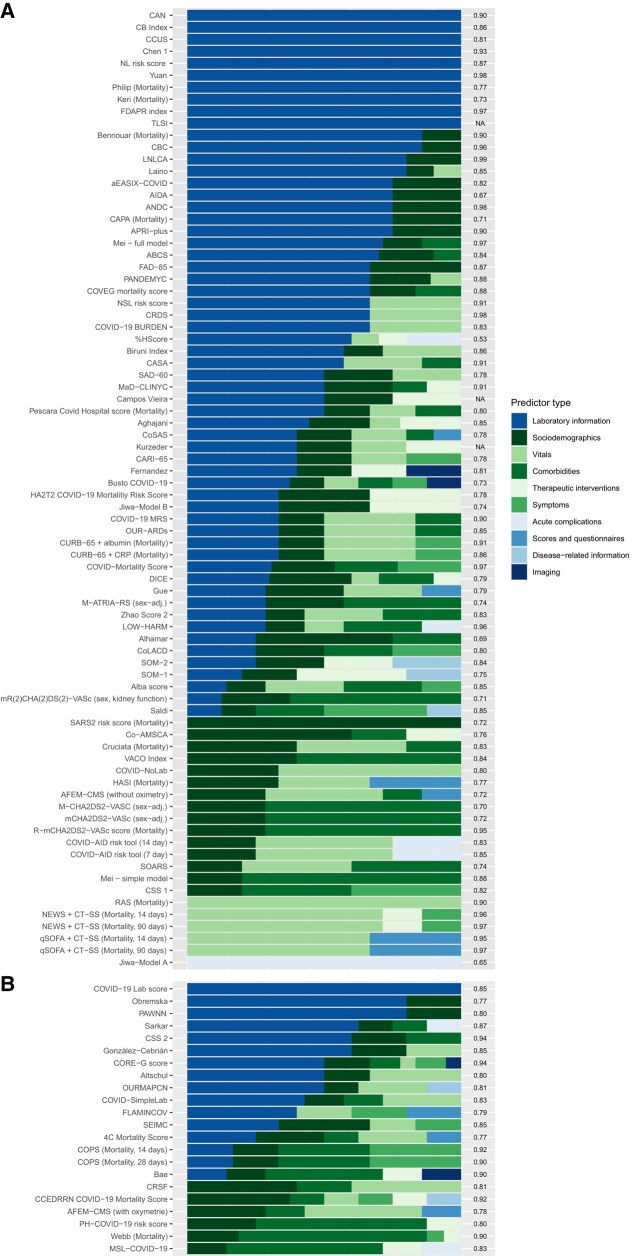
Predictor composition aggregated by predictor type for all scores assigned to category 1 stratified by the level of selection. *A*, Level 1, *B*, Level 2. The AUC is displayed on the right *y*-axis. The sorting of the scores is determined by (I) the absolute number of categories and (II) the relative proportion across all scores. The color gradient from green to blue indicates the availability of the category, although in case of doubt, this also depends on the level of care. Similar presentations of scores assigned to categories 2–4 are displayed in the Supplementary Material. Abbreviations: AUC, area under the curve; COVID-19, coronavirus disease 2019.

Among the 188 different predictors (extracted from all scores), age (68.2%) was the most frequently included, followed by C-reactive protein (CRP) (29.8%). This also applied to mortality or severity scores, where the importance of laboratory data, demographics, and physiological information stood out. Hospitalization scores often included age (87.8%) and dyspnea (57.1%) (for the top 20 predictors in each category, see Supplementary Table 3). The number of predictors per score ranged from two to 29. Figure 2 shows the frequency of predictor use in relation to the overall AUC of the scores. We also present the predictor domains by score, category, and inclusion level (Figure 3, Supplementary Figures 1 and 2).

### Characteristics of Scoring Systems Selected According to Predefined (Quality) Criteria (Level 2)

The most frequent outcomes among L2 scores were mortality as single endpoint (44.9%) or severity as composite outcome (36.7%). Among pure mortality and severity scores, 0.4%–51.2% and 3.7%–51.6% of the patients in the development cohorts reached the outcome, respectively. The estimated EPV ranged from 0.9 to 709.8 (eEPV < 10: 47.5%). The scores had a median AUC of 0.81 (IQR = [0.80, 0.87]).

Nine scores predicting hospitalization (18.4%) met the L2 criteria (outcomes: 4.0%–38.9%). The scores profited from larger sample sizes, with EPVs ranging from 15.6 to 120.7. The median AUC was 0.84 (IQR = [0.80, 0.85]).

The scores predicting PCC primarily used symptom information. None of them met the L2 criterium AUC ≥ 0.75 and were therefore not further analyzed.

### Risk of Bias

**Figure 4.**
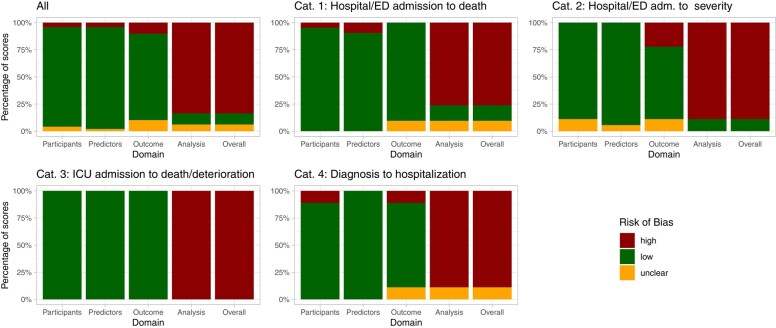
Results of the ROB assessment using PROBAST. Abbreviations: ED, emergency department; ICU, intensive care unit; ROB, risk of bias.

Many studies did not adhere to general guidance for developing predictive models [7, 15, 16], so that information relevant to their evaluation was unavailable. Most scores raised at least one concern within one of the PROBAST domains, leading to an overall high ROB (low 10.2%, unclear 6.1%, high 83.7%) (Figure 4, Supplementary Table 4). The primary concerns pertained especially to the “analysis” domain, namely, the absence of calibration measures (eg, adaptation of the relation of estimated and observed event probabilities) [17], failure to account for over-optimism (which could be met by, eg, bootstrapping or cross-validation) [15, 18], mishandling of missing values (eg, use of complete case analysis instead of imputation methods) [15], and insufficient validation techniques (eg, using random splits instead of geographical (external) validation) [15].

Table 4 shows scores with an overall ROB rating of “low” or “unclear.” Five scores were rated “low” ROB: (1) the ISARIC's 4C [12], (2) the CCEDRRN COVID-19 Mortality Score [20], (3) the SEIMC score [25] for the prediction of mortality, (4) the PRIEST score [23], and (5) the LMIC-PRIEST [21] for severity prognosis. Three more scores were rated with “unclear” ROB (AFEM [19], OURMAPCN [22], SARS2 [24]). These scores have common characteristics: their cumulative sample size was comparably large (except AFEM), and missing value imputation (except SEIMC) and calibration measures (except AFEM) were applied.

**Table 4.** Characteristics of Scores With Low or Unclear Risk of Bias (ROB) Rating

Score	Reference	Study Design	Population	Outcome	Year(s)^a^	Cumulative Sample Size; No. of Outcomes^b^	Final Predictors (No.; Description)	Strongest Type of Validation Reported	Performance of Strongest Type of Validation Reported AUC (95%-CI)	Overall ROB
4C Mortality Score	Knight et al [12]	Prospective observational cohort	Inpatients with “high likelihood” of COVID-19	Mortality (in-hospital)	2020	35 463 + 22 361; 11 426	n = 8: age, sex, number of comorbidities, RR, SaO2, GCS, urea level, CRP	Temporal validation with geographic subsetting	0.77 (.76–.77)	Low
AFEM-CMS—with SaO2	Pigoga et al [19]	Retrospective observational cohort	Inpatients with suspected, probable, or confirmed COVID-19	Mortality (in-hospital)	2020	374 + 93; 239*	n = 7; sex, age, number of comorbidities, GCS, systolic BP, RR, SaO2	Random split with cross-validation	0.78 (.74–.81)	Unclear
CCEDRRN COVID-19 Mortality Score	Hohl et al [20]	Retrospective observational cohort	ED patients with confirmed or suspected COVID-19	Mortality (in-hospital/ED)	2020–2021	6758 + 2054; 471	n = 8, age, sex, type of residence, arrival mode, chest pain, severe liver disease, RR, level of respiratory support	Geographical validation (same country, different centers)	0.92 (.90–.93)	Low
LMIC-PRIEST	Marincowitz et al [21]	Observational cohort study	ED patients with suspected or confirmed COVID	Mortality (in-hospital), intubation, NIV or ICU admission (30 d)	2020–2022	305 564 + 140 520 + 20 698; 12 610	n = 11; RR, SaO2, heart rate, systolic BP, temperature, alertness, inspired oxygen, sex, age, diabetes, heart disease	Geographical validation (other country)	0.79 (.79–.80)	Low
OURMAPCN	Chen et al [22]	Retrospective observational cohort	Inpatients with confirmed COVID-19	Mortality (in-hospital)	2020	6415 + 6351 + 2169 + 553; 462	n = 8; CRP, SpO2, admission date, age, BUN, RR, procalcitonin, neutrophils	Geographical validation (different country)	0.81 (.76–.86)	Unclear
PRIEST	Goodacre et al [23]	Retrospective observational cohort	Inpatients with suspected COVID-19	Death or organ support (30 d)	2020	11 773 + 9118; 2421	n = 9; age, sex, RR, systolic BP, SaO2/inspired oxygen ratio, performance status, consciousness, renal impairment, respiratory distress	Geographical validation (same country, different centers)	0.80 (.79–.81)	Low
SARS2 risk score	Dashti et al [24]	Retrospective observational cohort	Out- and inpatients with confirmed COVID-19	Hospitalization (30 d)	2020	10 496 + 1851; 3197	n = 5; age, sex, race, socioeconomic status, smoking	Validation with different population (medical staff)	0.77 (.73–.80)	Unclear
SEIMC	Berenguer et al [25]	Retrospective observational cohort	Inpatients with confirmed COVID-19	Mortality (30 d)	2020	4035 + 2126; 1047	n = 6; age, SaO2, NLR, GFR, dyspnea, sex	Temporal and geographical validation (same country, different centers)	0.85 (.82–.87)	Low

Further information on the selected set of scores and all scores assessed in Level 2 are presented in Supplementary Table 2. Abbreviations: AUC, area under the curve; BP, blood pressure; BUN, blood urea nitrogen; COVID-19, coronavirus disease 2019; CRP, C-reactive protein; DC, development cohort; ED, emergency department; GCS, Glasgow Coma Scale; GFR, glomerular filtration rate; RR, respiratory rate; NIV, non-invasive ventilation; NLR, neutrophils-lymphocytes-ratio; SaO2, oxygen saturation; VC, validation cohort.

^a^Recruitment year.

^b^Cumulative sample size consists of development cohort plus VC(s). Outcomes in the development cohort or the whole cohort (*) if not otherwise stated.

### External Validations

Only a fraction of the scores (n = 33) were validated externally (see Supplementary Table 5). The 4C was replicated most frequently: 37 validations from 20 countries yielded a primarily robust median AUC of 0.80 (range: 0.55 to 0.93) for different outcomes. Based on our literature search, most COVID-19 scores remained unvalidated.

## DISCUSSION

This systematic review investigated the quantity and quality of clinical scores predicting COVID-19 outcomes. Although numerous scores were developed specifically for this purpose, none were implemented in COVID-19 treatment guidelines [4, 11, 13] to become part of the clinical routine. Our analysis showed that most scores insufficiently adhered to the quality criteria required to ascertain validity, reliability, and trustworthiness.

### Scores Identified With Low or Unclear ROB

Most scores (n = 41) were found to carry a significant ROB due to methodological choices. We identified only 5 scores with low and 3 scores with unclear ROB (Table 4).

The 4C score [12] can be recommended for prognostication of mortality, as it is based on a large, prospective cohort, makes use of widely available predictors (in high-resource settings at hospital admission), and was frequently validated (at least 37 validations in 20 countries) [26]. Although the CCEDRRN COVID-19 Mortality [20] and SEIMC scores [25] were also developed on large cohorts, the designs of their development studies are based on retrospective data, and both would profit from additional validations (CCEDRRN: 0 validations; SEIMC: 4 validations, 4 countries). We identified the PRIEST score [23] and the LMIC-PRIEST [21], developed on large, multicenter cohorts, to be potentially suitable for the prognosis of COVID-19 severity. The LMIC-PRIEST might especially be relevant for low- and middle-income countries (LMIC) [21]. Although disease severity seems more relevant nowadays, only a few validation studies exist (PRIEST: 3 validations in 3 countries; LMIC-PRIEST: 0 validations, but external validation within the original study). A broad clinical application should thus wait for further validation. In general, an adjustment to altered frequencies of COVID-19-related deaths and severe disease courses since score development (eg, by immunity and vaccination) should be investigated.

### Predictor Selection, Applicability, and Complexities

Many scores included a wide range of predictors (Figure 2) from different domains (Figure 3). We may assume that data availability often impacts the predictor choices more than what is recommended by best practice guidelines [6, 27]. This heterogeneity is most likely a result of differences across studies, such as the scope of data sources used, entry criteria for analyzed cohorts, slight differences in endpoints and definitions, and statistical approaches employed. The heterogeneous clinical appearance of COVID-19 and changing vaccination statuses may have added to that heterogeneity. However, the review reveals a common set of predictors used in many scores (eg, age and CRP), whereas others were included in only 1 or very few (eg, nausea or hypotension).

Our results indicate that COVID-19 mortality or severity scores should include age, respiratory conditions, laboratory data, and comorbidities to predict outcomes reliably [10, 28]. Pre-hospital scores (eg, predicting hospital admission) primarily use information on comorbidities and sociodemographic information, applicable without diagnostic infrastructure. Overall, symptoms and imaging appeared to play a minor role. Among the 20 most frequently used predictors, 6 (age, sex, diabetes, hypertension (as part of metabolic syndrome), blood urea nitrogen (BUN), creatinine) represent components of baseline assessment for (organ-related) infection outcomes or differential diagnoses, 10 (CRP, lactate dehydrogenase, oxygen saturation, respiratory rate, neutrophils-lymphocyte ratio, lymphopenia, dyspnea, thrombocytopenia, blood pressure, paO2/FiO2, temperature, and leukocytes) are accepted markers of overall infection severity/sepsis [29–32]. In contrast, only 2 (D-dimer, albumin) may not be universally accepted as part of a baseline assessment for moderate to severe respiratory illnesses [33, 34]. It is not surprising that studies primarily confirm prior knowledge, since clinical practice is based on existing evidence. This in turn leads to selection of established markers for patient screening and thus limited availability of markers for score validation. Prospective determination of comprehensive metabolic panels might well lead to more effective models. This observation may partly be attributed to prior knowledge as a key criterion in defining the data sets that, in turn, were used in the analyses. It should be emphasized this means that the most used criteria are generally available during patient care in medium to high-resource settings. However, given the considerable overlap of predictors of general infection and severity/sepsis, it also suggests that the scores might not add much to existing knowledge on respiratory infection outcomes and are probably not very specific to COVID-19.

Non-routine laboratory indicators such as D-dimer and interleukine-6 restrict score applicability to high-resource settings [10]. However, because D-dimer belonged to the top 10 predictors (Figure 2), further studies are warranted to define the incremental value of such parameters for successful patient management. Overall, laboratory tests for scores (eg, indicators of kidney function or protein metabolism [urea, BUN, creatinine] or indicators for inflammation such as the CRP vs leucocytes) may restrict practicability to specific resource and management settings. Not all general practitioners and outpatient departments will perform comprehensive tests based on moderate respiratory symptoms [35]. The association between data availability, care setting, and regional standards is a likely source of bias that may limit transferability. Furthermore, non-conventional or time-dependent predictors such as arrival mode [20] or admission date [22] are less generalizable for validation in most cohorts. Only one score asked for the vaccination status [36], as most scores were developed on data from the early pandemic. Results from clinical trials indicate that vaccination status may be among the most critical outcome predictors today [37, 38].

### Limitations of the Evidence Included in the Review

Differences in score development design may lead to varied performance [15]. Notably, we observed a substantial variation in sample sizes, settings, and case definitions. Population characteristics, including age [28, 39], ethnicity [40], and immunity influence COVID-19 outcomes. Additionally, we noted differences in preconditions for specific therapies or hospital admissions among countries [41]. Further complicating matters, the comparability of composite outcomes was limited due to the variation in the combination selected by different study groups.

Good performance measures, in combination with small sample sizes or inconsistent reporting of both discrimination and calibration measures, indicate a higher risk for overfitting [15, 42]. Regarding “all” scores, high AUCs (78.5% ≥ 0.75) often came together with relatively small sample sizes (64.0% ≤ 1000 patients). Scores should not be applied in clinical practice until validations show generalizability, applicability, and robust performances across various patient characteristics that match regional circumstances [8, 42, 43].

### Comparison to Other Studies

With the abundance of published models and scores, identifying “all” relevant items is demanding. Therefore, complementary approaches are needed. We identified a few reviews on COVID-19 predictions or scores, all focusing on different approaches and yielding a (slightly) different set of models, both overall and in terms of low ROB [8, 9, 44, 45]. The 4C score [12], the PRIEST model [23] and the NEWS2 were repeatedly discussed as favorable prognostic tools.

### Limitations of the Review Process

We restricted the detailed analysis to scores that fulfilled predefined criteria; thus, the L2 results refer to scores not representative of “all” scores. A broader approach, including additional sources, might have revealed further relevant studies. We did not contact the studies' authors for missing information and used a restricted *Checklist for critical Appraisal and data extraction for systematic Reviews of prediction Modelling Studies* (CHARMS) checklist [7] (see Supplementary Text 3) focused on aspects considered most relevant to our research question. Well-established early warning scores were not within our scope but are reported to have a robust performance in validation studies [46].

## CONCLUSION

Our study is a comprehensive analysis of COVID-19 scores regarding predictor assessment and applicability. Most scores exhibited a marked ROB and lacked external validation. In future pandemics, data and resource sharing alongside the application of recommended model development and reporting guidelines [6, 7, 15, 16] would improve score quality and visibility, leading to better implementation for the benefit of the patients.

With currently 3 years of COVID-19 investigations at data retrieval, we also recognize the absence of reliable scoring systems for the prognosis of PCC. Because outcomes have continuously improved since the first wave of the pandemic, many experts consider PCC has surpassed severe illness and death as a health hazard. Reliable predictors of poor long-term outcomes would be an asset for decision making and the design of future clinical trials.

In conclusion, none of the numerous scores that have been developed received strong guideline recommendations on an international level. The current consensus is that the predictive tools for COVID-19 are helpful but should only support and not replace physician's judgments.

## Supplementary Data

Supplementary materials are available at *Clinical Infectious Diseases* online. Consisting of data provided by the authors to benefit the reader, the posted materials are not copyedited and are the sole responsibility of the authors, so questions or comments should be addressed to the corresponding author.

## Supplementary Material

ciad618_Supplementary_Data

## Notes

***Author Contributions.*** J. J. V. conceptualized, and J. J. V. and K. A. designed the study. K. A. and R. G. performed the systematic review including title, abstract, and full text screening and the PROBAST assessment in duplicate. K. A., D. M., R. G., O. M., and J. J. V. interpreted the findings. J. J. V. and S. M. H. supported with clinical knowledge regarding the content of the review. K. A. drafted the manuscript, and all authors critically reviewed all materials and approved the final manuscript.

***Financial support*.** This work was supported by ORCHESTRA. The ORCHESTRA project has received funding from the European Union’s Horizon 2020 research and innovation program under grant agreement No 101016167. The views expressed in this publication are the sole responsibility of the author, and the Commission is not responsible for any use that may be made of the information it contains. This publication was supported by the German Federal Ministry of Education and Research (BMBF) Network of University Medicine 2.0: “NUM 2.0” (grant number 01KX2121), Project: National Pandemic Cohort Network (NAPKON).
