# Supplementary material for: A Systematic Review of Predictor Composition, Outcomes, Risk of Bias, and Validation of COVID-19 Prognostic Scores
Source: Clin Infect Dis. 2023 Oct 25;78(4):889–99. doi: 10.1093/cid/ciad618 (PMC11006104; doi:10.1093/cid/ciad618)
Supplement: ciad618_Supplementary_Data [file ciad618_supplementary_data.docx]

# Supplementary material to

**A Systematic Review of Predictor Composition, Outcomes, Risk of Bias, and Validation of COVID-19 Prognostic Scores**

**Supplementary Text 1.** Reasons for the selection criteria (Level 2).

We chose the AUC as the primary performance metric. Although discrimination measures alone are insufficient, it is a minimum requirement for a qualitative model[1]. We included scores with AUCs ≥ 0.75 with a "clearly useful discrimination"[1] and reported the performance measure estimated from the strongest validation reported (external validation (different centers, countries, populations) > temporal validation > random split > development and validation in the exact same data set)[2,3]. A validation cohort is a mandatory quality criterion for the development of clinical prediction rules since a model usually performs better in the cohort it was developed on[4,5]. In addition, a multicenter - thus more heterogenous - setting increases the chance of transferability. To support clinical and independent applicability, we added a point-based calculation as a criterion and excluded studies with (sophisticated) formulas to minimize required resources in a strained clinical environment.

**Supplementary Text 2.** Search strategy in PubMed/MEDLINE and Web of Science

**#1 AND #2 AND #3 AND #4**

**#1 COVID-19**

| **PubMed** | **Web of Science** |
| --- | --- |
| ("COVID-19"[Mesh] OR "SARS-CoV-2"[Mesh] OR “COVID-19”[tiab] OR “COVID19”[tiab] OR “SARS-CoV-2 Infectio*”[tiab] OR “2019 Novel Coronavirus”[tiab] OR “2019-nCoV Diseas*”[tiab] OR “2019-nCoV Infectio*”[tiab] OR “COVID-19 Virus Diseas*”[tiab] OR “COVID-19 Virus Infectio*”[tiab] OR “Coronavirus Disease 2019”[tiab] OR “Severe Acute Respiratory Syndrome Coronavirus 2 Infectio*”[tiab] OR (("coronavirus"[MeSH Terms] OR "coronavirus"[tiab] OR "COV"[tiab]) AND 2019/11/01[PDAT] : 2023/12/31[PDAT])) | TS=(“COVID-19” OR COVID19 OR “SARS-CoV-2 Infectio*” OR “2019 Novel Coronavirus” OR “2019-nCoV Diseas*” OR “2019-nCoV Infectio*” OR “COVID-19 Virus Diseas*” OR “COVID-19 Virus Infectio*” OR “Coronavirus Disease 2019” OR “Severe Acute Respiratory Syndrome Coronavirus 2 Infectio*”) OR (TS=(coronavirus OR COV) AND (DOP=(2019-11-01/2023-12-31))) |

**#2 Prediction**

| **PubMed** | **Web of Science** |
| --- | --- |
| ("forecas*"[tiab] OR “stratif*”[tiab] OR “prognos*”[tiab] OR "predic*"[tiab] OR "risk assessment"[tiab]) | TS=(forecas* OR stratif* OR prognos* OR predic* OR “risk assessment”) |

**#3 Scoring**

| **PubMed** | **Web of Science** |
| --- | --- |
| ("Clinical Decision Rules"[Mesh] OR "scor*"[title] OR "index"[title] OR "indices"[title] OR "scal*"[title] OR "clinical decision rul*"[title] OR "tool*"[title] OR "algorith*"[title] OR “clinical signature”[title]) | TI=(scor* OR index OR indices OR scal* OR “clinical decision rul*” OR tool* OR algorith* OR “clinical signature”) |

**#4 Validation** **Metrics**

| **PubMed** | **Web of Science** |
| --- | --- |
| ("Area Under Curve"[Mesh] OR "ROC Curve"[Mesh] OR "Sensitivity and Specificity"[Mesh] OR "Data Accuracy"[Mesh] OR “validat*”[tiab] OR “discriminat*”[tiab] OR “calibrat*”[tiab] OR “AUC”[tiab] OR “AUCs”[tiab] OR “AUROC”[tiab] OR “ROC”[tiab] OR “area under the”[tiab] OR “receiver operating characteristi*”[tiab] OR “sensitivity”[tiab] OR “specificity”[tiab] OR “coefficient of determination”[tiab] OR “incidence”[tiab] OR “accuracy”[tiab] OR “negative predictive value”[tiab] OR “positive predictive value”[tiab] OR “NPV”[tiab] OR “PPV”[tiab] OR “c-statisti*”[tiab] OR “false-positive”[tiab] OR “false-negative”[tiab] OR “false discovery rate”[tiab]) | TS=(validat* OR discriminat* OR calibrat* OR AUC OR AUCs OR AUROC OR ROC OR “area under the” OR “receiver operating characteristi*” OR sensitivity OR specificity OR “coefficient of determination” OR incidence OR accuracy OR “negative predictive value” OR “positive predictive value” OR NPV OR PPV OR “c-statisti*” OR “false-positive” OR “false-negative” OR “false discovery rate”) |

The results of both databases were merged using the Digital Object Identifier (DOI). No further filter criteria were applied.

**Supplementary Text 3.** Data extraction items.

Data for all scores were extracted on the following items:

Name of the score, first author, year, title, study design, number of participating centers, heath care level, sample size (development cohort, validation cohort), population, age, country of derivation, timing of predictor measurement, primary outcome(s), outcome events (in the development cohort if stated), number of predictors, predictors, type of combination of predictors, separated validation cohort and the area under the receiver operating characteristic curve (AUC/AUROC). We reported on performance statistics (AUC) as stated from the strongest form of validation available (see S1).

Additional data for the selected set of scores (Level 2) were extracted or derived from the following items based on the CHARMS checklist[6] and PROBAST guidelines[5]:

Study design (additional information), inclusion and exclusion criteria, study dates, definition and method for measurement of outcome, time of outcome occurrence or summary of duration of follow-up, number of candidate predictors (self-counted if not precisely stated; indicated by “~”), handling of continuous variables, events-per-variable, number of participants with any missing value, handling of missing values, modelling method, selection method of final predictors, shrinkage of predictor weights or regression coefficients/account for overfitting and optimism, complexities in the data, calibration measures, classification measures, method used for testing model performance.

While the absolute sample size is a first indication of generalizability, is not a suitable criterion for the sole assessment of the study power. The number of outcomes in relation to the number of candidate predictors (events-per-variable) is more adequate. Even though the number of regression coefficients would be more precise, we derived the absolute number of candidate predictors used during model development as approximation. Events-per-Variable (EPV) should not fall below 10 (better: 20), otherwise indicating a higher risk for overfitting of the model[5].

**Supplementary Table 1.** Characteristics of all included scores.

Download the Excel file here: https://cloud.idcohorts.net/s/AHNnNnXarYa6SwE

**Supplementary Table 2.** Characteristics of the set of scores included in Level 2.

Download the Excel file here: https://cloud.idcohorts.net/s/dg6MxEjt8k4nP58

**Supplementary Table 3.** Ranking of TOP 20 predictors by category.

| **#** | **All scores** | **n** | **%** | **Category 1** | **n** | **%** | **Category 2** | **n** | **%** |
| --- | --- | --- | --- | --- | --- | --- | --- | --- | --- |
| 1 | Age | 165 | 68.2 | Age | 77 | 77.0 | Age | 67 | 59.8 |
| 2 | C-reactive protein | 72 | 29.8 | C-reactive protein | 28 | 28.0 | C-reactive protein | 40 | 35.7 |
| 3 | Oxygen saturation | 63 | 26.0 | Oxygen saturation | 28 | 28.0 | Lactat dehydrogenase | 36 | 32.1 |
| 4 | Sex | 58 | 24.0 | Sex | 26 | 26.0 | Oxygen saturation | 31 | 27.7 |
| 5 | Lactat dehydrogenase | 54 | 22.3 | Respiratory rate | 21 | 21.0 | Lympocytes/lymphopenia | 27 | 24.1 |
| 6 | Respiratory rate | 45 | 18.6 | Lactat dehydrogenase | 17 | 17.0 | Sex | 23 | 20.5 |
| 7 | Lympocytes/lymphopenia | 45 | 18.6 | Lympocytes/lymphopenia | 17 | 17.0 | Respiratory rate | 22 | 19.6 |
| 8 | Diabetes mellitus (T2 and/or T1) | 39 | 16.1 | D-Dimer | 17 | 17.0 | D-Dimer | 20 | 17.9 |
| 9 | D-Dimer | 38 | 15.7 | Neutrophils-lymphocytes ratio | 16 | 16.0 | Diabetes mellitus (T2 and/or T1) | 20 | 17.9 |
| 10 | Dyspnea/respiratory distress | 36 | 14.9 | Thrombocytes/thrombocytopenia | 16 | 16.0 | Dyspnea/respiratory distress | 18 | 16.1 |
| 11 | Neutrophils-lymphocytes ratio | 34 | 14.0 | Diabetes mellitus (T2 and/or T1) | 14 | 14.0 | Neutrophils-lymphocytes ratio | 16 | 14.3 |
| 12 | Blood pressure | 30 | 12,4 | Blood pressure | 14 | 14.0 | Blood pressure | 13 | 11.6 |
| 13 | Thrombocytes/thrombocytopenia | 28 | 11.6 | Urea/ Blood urea nitrogen | 13 | 13.0 | Albumin | 13 | 11.6 |
| 14 | Urea/ Blood urea nitrogen | 27 | 11.2 | Leukocytes | 13 | 13.0 | Temperature | 12 | 10.7 |
| 15 | Hypertension | 26 | 10.7 | Creatinine | 12 | 12.0 | paO2/FiO2 | 11 | 9.0 |
| 16 | Albumin | 24 | 9.9 | Hypertension | 10 | 10.0 | Urea/ Blood urea nitrogen | 10 | 8.9 |
| 17 | paO2/FiO2 | 23 | 9.5 | Albumin | 10 | 10.0 | Hypertension | 10 | 8.9 |
| 18 | Creatinine | 21 | 8.7 | (Un)conciousness/confusion/alertness | 10 | 10.0 | Neutrophils | 10 | 8.9 |
| 19 | Temperature | 19 | 7.9 | Dyspnea/respiratory distress | 9 | 9.0 | Thrombocytes/thrombocytopenia | 9 | 8.0 |
| 20 | Leukocytes | 19 | 7.9 | Neutrophils | 9 | 9.0 | Body Mass Index | 8 | 7.1 |
| **#** | **Category 3** | **n** | **%** | **Category 4** | **n** | **%** | **Category 5** | **n** | **%** |
| 1 | Age | 9 | 69.2 | Age | 12 | 85.7 | Sex | 1 | 33.3 |
| 2 | Vasopressors or mean arterial pressure | 5 | 38.5 | Dyspnea/respiratory distress | 8 | 57.1 | Number of symptoms | 1 | 33.3 |
| 3 | Urea/ Blood urea nitrogen | 4 | 30.8 | Sex | 6 | 42.9 | CD24+CD38lo/- B cells | 1 | 33.3 |
| 4 | pH | 4 | 30.8 | Hypertension | 6 | 42.9 | Naive CD4+ T cells | 1 | 33.3 |
| 5 | Blood pressure | 3 | 23.1 | Presence of comorbidities | 4 | 28.6 | Fluoresc. of CD57 in CD8+ T cells | 1 | 33.3 |
| 6 | Temperature | 3 | 23.1 | Diabetes mellitus (T2 and/or T1) | 4 | 28.6 | Acute phase symptoms (n=27) | 1 | 33.3 |
| 7 | paO2/FiO2 | 3 | 23.1 | paO2/FiO2 | 3 | 21.4 | Post-COVID symptoms (n=29) | 1 | 33.3 |
| 8 | Glasgow Coma Scale | 3 | 23.1 | Chronic kidney injury | 3 | 21.4 |  |  |  |
| 9 | Oxygen index/oxygenation | 3 | 23.1 | Fever | 3 | 21.4 |  |  |  |
| 10 | C-reactive protein | 2 | 15.4 | Chronic respiratory disease | 3 | 21.4 |  |  |  |
| 11 | Oxygen saturation | 2 | 15.4 | Ethnicity | 3 | 21.4 |  |  |  |
| 12 | Sex | 2 | 15.4 | C-reactive protein | 2 | 14.3 |  |  |  |
| 13 | Thrombocytes/thrombocytopenia | 2 | 15.4 | Oxygen saturation | 2 | 14.3 |  |  |  |
| 14 | Creatinine | 2 | 15.4 | Immunosuppression | 2 | 14.3 |  |  |  |
| 15 | Chronic kidney injury | 2 | 15.4 | Chronic artery disease | 2 | 14.3 |  |  |  |
| 16 | Leukocytes | 2 | 15.4 | Fatigue | 2 | 14.3 |  |  |  |
| 17 | Ferritin | 2 | 15.4 | Chronic liver disease | 2 | 14.3 |  |  |  |
| 18 | Presence of comorbidities | 2 | 15.4 | Residence | 2 | 14.3 |  |  |  |
| 19 | Lactate | 2 | 15.4 | Congenital heart failure | 2 | 14.3 |  |  |  |
| 20 | (Sa)FiO2 | 2 | 15.4 | Temperature | 1 | 7.1 |  |  |  |

We present absolute (n) and relative (%) amounts of the respective predictors included in the evaluated scores, stratified by category. For the category definitions see **Table 2**.

**Supplementary Table 4**. PROBAST results by score and domain.

| **Name Score** | **Participants** | **Predictors^a^** | **Outcome** | **Analysis** | **Overall** |
| --- | --- | --- | --- | --- | --- |
| 4C Mortality Score | low | low | low | low | low |
| Aboumrad | low | low | low | high | high |
| AFEM COVID-19 Mortality Scale (AFEM-CMS) - with oxymetrie | low | low | low | unclear | unclear |
| Altschul | low | low | low | high | high |
| Bae | low | low | low | high | high |
| BAS^2^IC Score | low | low | low | high | high |
| CALL (Comorbidity, Age, Lymphocyte, LDH) | low | low | high | high | high |
| CAPS-D score (critical illness, 7 days) | low | low | low | high | high |
| CAPS-D score (critical illness, complete FU) | low | low | low | high | high |
| CCEDRRN COVID-19 Mortality Score | low | low | low | low | low |
| Chen 2 | low | low | high | high | high |
| CHOSEN (COVID Home Safely Now) | low | unclear | high | high | high |
| COPS (COVID-19 Prognosis Score) - Mortality (14 days) | low | low | low | high | high |
| COPS (COVID-19 Prognosis Score) - Mortality (28 days) | low | low | low | high | high |
| CORE-G score | low | low | low | high | high |
| COSA | low | low | low | high | high |
| COVIC | low | low | low | high | high |
| COVID-NoLab | low | high | low | high | high |
| COVID-SimpleLab | low | high | low | high | high |
| CRSF (COVID-19 Risk-Score in Fars Province) | high | low | low | high | high |
| CSS (Scoring System of COVID-19) | low | low | low | high | high |
| Ebell - Model A | low | low | low | high | high |
| Ebell - Model B | low | low | low | high | high |
| Ebell - Model C | low | low | low | high | high |
| Ebell - Model D | low | low | low | high | high |
| FLAMINCOV score | low | low | low | high | high |
| González-Cebrián | low | low | low | high | high |
| HOPE Sepsis Score | low | low | unclear | high | high |
| Huang | low | low | low | high | high |
| Linssen | low | low | low | high | high |
| LMIC-PRIEST | low | low | low | low | low |
| MCC19-RS (Mayo Clinic COVID-19 risk score) | high | low | low | high | high |
| MMCD score | low | low | high | high | high |
| MSL-COVID-19 | low | low | unclear | high | high |
| Obremska | low | low | low | high | high |
| OURMAPCN-score | low | low | low | unclear | unclear |
| PAWNN score | low | low | low | high | high |
| PH-(Patient History) COVID-19 risk score | low | low | low | high | high |
| PRIEST | low | low | low | low | low |
| qCSI (quick Covid-19 Severity Index) | low | low | high | high | high |
| RECOILS (Rapid Evaluation of Coronavirus Illness Severity score) | low | low | low | high | high |
| Sarkar | low | low | unclear | high | high |
| SARS2 risk score (Hospitalization) | low | low | low | unclear | unclear |
| SEIMC | low | low | low | low | low |
| SIMI score | low | low | low | high | high |
| Webb (Hospitalization) | low | low | unclear | high | high |
| Webb (Mortality) | low | low | unclear | high | high |
| Yamada (age 18-39 years) | unclear | low | low | high | high |
| Yamada (age 40-64 years) | unclear | low | low | high | high |

We performed the analysis according to PROBAST guidelines. A “low” risk of bias rating for a model or study indicates that the confidence in the study is higher and the reader can draw stronger conclusions from the respective study compared to a study with “high” risk of bias.[5] "No information" if no information was provided. If we rated 2.2 as “no information” we allocated a low overall rating for the predictor domain, to acknowledge the little ROB for objective predictors, where applicable.

**Supplementary Table 5.** Overview of external validations of the scores included in this review.

The collection of external validation studies was performed using an ad hoc search in the same literature retrieval and a reduced data extraction form compared to the original studies. The data extraction was not performed in duplicate (KA).

Download the Excel file here: <https://cloud.idcohorts.net/s/R26bZoR2MCS5nxq>


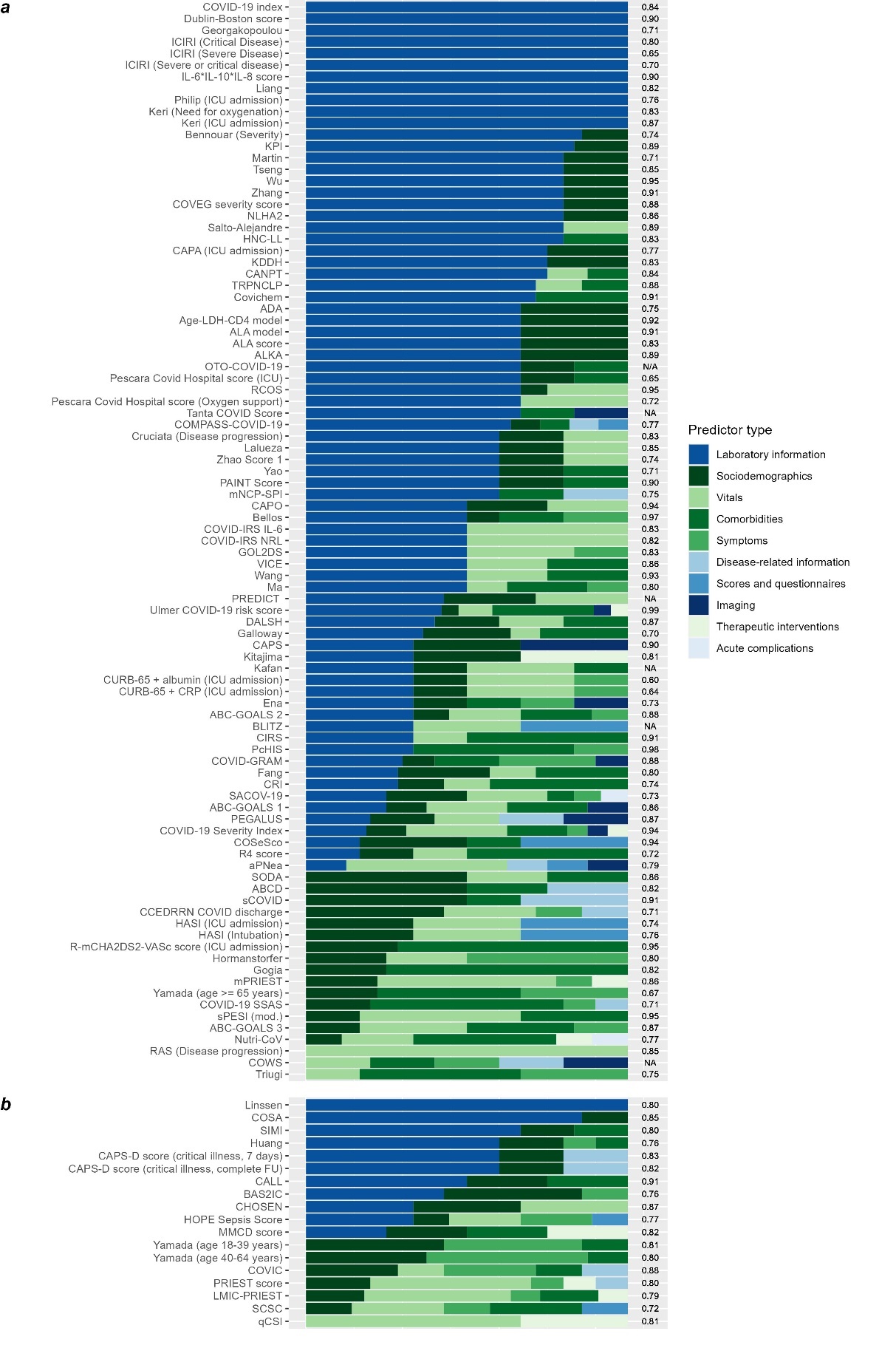


**Supplementary Figure 1.** Predictor composition aggregated by predictor type for scores assigned to category 2.

Subfigure a and b correspond to scores within category 2 assigned to Level 1 and 2, respectively. The sorting of the scores is determined by (I) the absolute number of categories and (II) the relative proportion across all scores. The color gradient from green to blue indicates the availability of the category, although in case of doubt this also depends on the level of care.


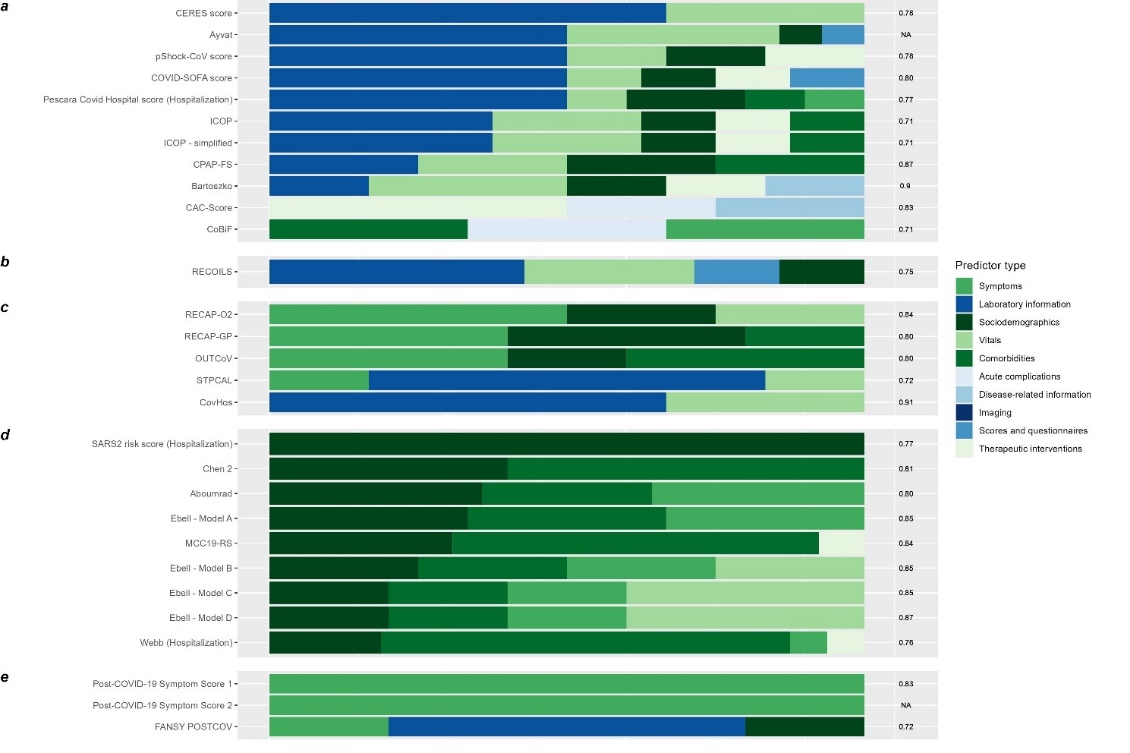


**Supplementary Figure 2.** Predictor composition aggregated by predictor type for scores assigned to category 3, 4 and 5.

Subfigures a, b, c, d and e correspond to category 3 (L1), 3 (L2), 4 (L1), 4 (L2), and 5, respectively. The sorting of the scores is determined by (I) the absolute number of categories and (II) the relative proportion across all scores. The color gradient from green to blue indicates the availability of the category, although in case of doubt this also depends on the level of care.

**References**

1. Alba AC, Agoritsas T, Walsh M, et al. Discrimination and Calibration of Clinical Prediction Models: Users' Guides to the Medical Literature. JAMA **2017**; 318:1377–84.

2. Ramspek CL, Jager KJ, Dekker FW, Zoccali C, van Diepen M. External validation of prognostic models: what, why, how, when and where? Clin Kidney J **2021**; 14:49–58.

3. Wynants L, van Calster B, Collins GS, et al. Prediction models for diagnosis and prognosis of covid-19: systematic review and critical appraisal. BMJ **2020**; 369:m1328.

4. Cowley LE, Farewell DM, Maguire S, Kemp AM. Methodological standards for the development and evaluation of clinical prediction rules: a review of the literature. Diagn Progn Res **2019**; 3:16.

5. Moons KGM, Wolff RF, Riley RD, et al. PROBAST: A Tool to Assess Risk of Bias and Applicability of Prediction Model Studies: Explanation and Elaboration. Ann Intern Med **2019**; 170:W1-W33.

6. Moons KGm, Groot JAH de, Bouwmeester W, et al. Critical appraisal and data extraction for systematic reviews of prediction modelling studies: the CHARMS checklist. PLoS Med **2014**; 11:e1001744.
